# Towards a conceptual model for the use of home healthcare medical devices: The multi-parameter monitor case

**DOI:** 10.1371/journal.pone.0208723

**Published:** 2018-12-07

**Authors:** Pablo Reyes, Dominique Larée, Alejandro Weinstein, Álvaro Jara

**Affiliations:** 1 School of Biomedical Engineering, Universidad de Valparaíso, Valparaíso, Chile; 2 Advanced Center for Electrical and Electronics Engineering, Universidad Técnica Federico Santa María, Valparaíso, Chile; Fondazione Ugo Bordoni, ITALY

## Abstract

In the last decade there has been an increase in the use of medical devices in the home environment. These devices are commonly the same as those used in hospitals by healthcare professionals. The use of these these devices by lay users outside of a clinical environment may become unsafe. This study presents a methodology that allows decision makers to identify potential risk situations that may arise when lay users operate healthcare medical devices at home. Through a usability study based on the Grounded Theory methodology, we create a conceptual model in which we identified problems and errors related to the use of a multi-parameter monitor in a home environment by a group of lay users. The conceptual model is reified as a graphical representation, which allows stakeholders to identify (i) the weaknesses of the device, (ii) unsafe operation modes, and (iii) the most suitable device for a specific user.

## Introduction

A medical device is any instrument, machine, software, or other similar equipment intended to manage human diseases and injuries, support human life or examine specimens, among other uses [1]. The user of a medical device is usually a professional in a healthcare facility. A Home Healthcare Medical Device (HHMD) is a medical device intended for use at home without the need of specialists to operate them and dedicated to improve the patient’s quality of life [2].

The use of HHMD is a fast growing sector in the health care industry [3–5]. The Global Home Healthcare Market is expected to reach USD 303,605.9 million by 2020, growing at a Compound Annual Growth Rate (CAGR) of 8.1% from 2014 to 2020 [6]. The most significant drivers for this increasing in use are: (1) the increasing aging population [4, 7] and their associated chronic diseases. Continua Health Alliance establishes that 860 million people have one or more chronic conditions [8]; and (2) the motivation for health institutions to reduce medical cost by decreasing the length of hospital stays and redirecting early patients to home care [9].

A medical device must be designed to ensure safety and effectiveness. Safety is achieved by reducing the risks associated with user error as far as possible. Effectiveness is achieved when the performance intended by the manufacturer is realized, and the device is suitable for the intended purpose [10]. However, HHMDs are required to ensure this condition of safety and effectiveness while being operated by a lay user in a home environment [11].

Lay operators are generally non-specialists, and are not always trained adequately in the use of medical equipment [7, 12, 13]. Additionally, these users may have limitations and disabilities [7, 12], and form a heterogeneous group which is difficult to profile [12–14].

With regards to the nonclinical environment, the IEC 60601-1-11 (2010) standard defines the home healthcare environment as the place where a patient lives, or other places where patients are present (excluding health centers where health professionals are continuously available) [15]. In this environment, common risks in health care are also prevalent. However, there are additional risks inherent to the home, related to geographic location, construction, home maintenance, and other unsafe conditions [7, 9].

Several standards regulate the design of HHMDs: The ANSI/AAMI HE75:2009 (2009) standard regulates the design of HHMDs from a human factor engineering point of view. The IEC 60601-1-11:2010 defines the requirements for basic safety and essential performance for medical devices used in the home healthcare environment [16]. Furthermore, the Food and Drug Administration has cleared HHMDs to be used outside of clinical settings once the required safety and effectiveness have been certified [5]. The Food and Drug Administration (FDA) launched the ‘Medical Device Home Use Initiative’ in 2010. The aim of this initiative is to assist manufacturers in designing and developing home use devices that comply with applicable standards of safety and effectiveness, along with other regulatory requirements [5].

In spite of these regulations, medical devices and equipment originally designed for use in hospitals are increasingly found in the home environment [3, 6, 9, 17]. Furthermore, older medical devices—which have been replaced by newer versions in health care institution—migrate to the home environment due to their increased availability [3, 13]. As such, the users may not receive the appropriate training or customer support required to use the device appropriately [13, 17]. These known issues lead to the potentially unsafe operation of medical devices in a home environment [17, 18]. This issue is so prominent that the Emergency Care Research Institute (ECRI) established the use of medical devices at home as one of the top ten technology hazards for 2012 [19].

Furthermore, the current research on patient safety focuses mainly on hospitals, where many initiatives have been established to reduce preventable injuries and deaths [18]. There appears to be considerably less research and patient safety initiatives in other healthcare sectors such as in the home. Although some studies have helped to understand and address some of the new challenges imposed by using medical devices at home [2, 7, 17], more studies are necessary to evaluate the impact of the usually non-optimal operational conditions of medical device at home [7, 14].

The risks associated with the operation of a device can be demonstrated by a usability test. Usability testing allows the evaluation of effectiveness, efficiency, and satisfaction that a user achieves while performing a set of tasks [20]. Usability tests were initially used to asses software user interfaces [21, 22], and are also currently applied in the healthcare sector in the design of health information systems and medical devices [23–26]. One of the first stages of a usability engineering process and risk management (related to the IEC 62366 (2007) and ISO 14971) is to identify the hazards, hazardous situations, and characteristics related to safety [27]. However, these standards do not detail a method or technique to identify hazardous situations, or to determine characteristics related to safety. In 2016, the FDA released the guide “Applying Human Factors and Usability Engineering to Medical Devices” [28]. This guide establishes the application of human factors engineering (HFE) and usability engineering (UE) concepts to reduce the risk and to ensure “that devices are safe and effective for the intended users, uses, and use environments.” The guide focuses on managing the risks associated with the use of medical devices across the HFE/UE concepts, and it is based on a model of the interactions between a user and a device. This model considers: (1) information perceived by the user, (2) cognitive processing, and (3) control actions. The objective of this guide is to help manufacturers to improve the design of the devices.

This work focuses on the new risks that arise when a medical device designed for a clinical environment is used at home. In particular, the objective of this work is to generate a methodology that aids in the understanding of these risks through a case study of the usability of a multi-parameter monitor device in a home care environment. Applying this methodology, we will develop a conceptual model in which the characteristics related to safety and hazardous situations emerge. This conceptual model is based on observations of lay users operating the medical device in their home environment.

### Grounded theory

Grounded theory method (GT) is a systematic methodology that involves the discovery of theory through the analysis of data of a given phenomenon. In GT, the conceptual framework (a hierarchical and coherent classification of concepts) emerges through the continuous interaction between data collection and data analysis. That is, a theory emerges inductively from the data [29].

In this work we perform a usability case study using this methodology. The data is gathered through the observation of the use of a multi-parameter monitor (designed for a clinical environment) operated by a non-healthcare professional. The collected data is then analyzed according to the Grounded Theory methodology [30]. This methodology allows to structure and to determine a hierarchy of concepts that emerges from the use of the medical device. For example, through the use of this methodology we will determine that the concept “User does not know if he is satisfied with the selected alarm level because he cannot test it” emerges from volunteer comments such as: “How do I know which sound level is appropriate to me? I cannot hear it”, “Will this sound level be enough? Can I test it?” or “I cannot know how loud it is, so I will put a high number to be sure to hear it well”.

We focused on identifying problems and errors emerging during the operation of a medical device by lay users in order to obtain a conceptual model of the use of medical devices in a homecare environment. This study uses a medical device and not a HHMD, because in practice medical devices originally designed for clinical use are increasingly being used in the home environment [9]. Testing in the real operational environment—homecare with the device operated by a lay user—can reveal problems that have not been anticipated either by the standards or designers [14].

**Fig 1:**
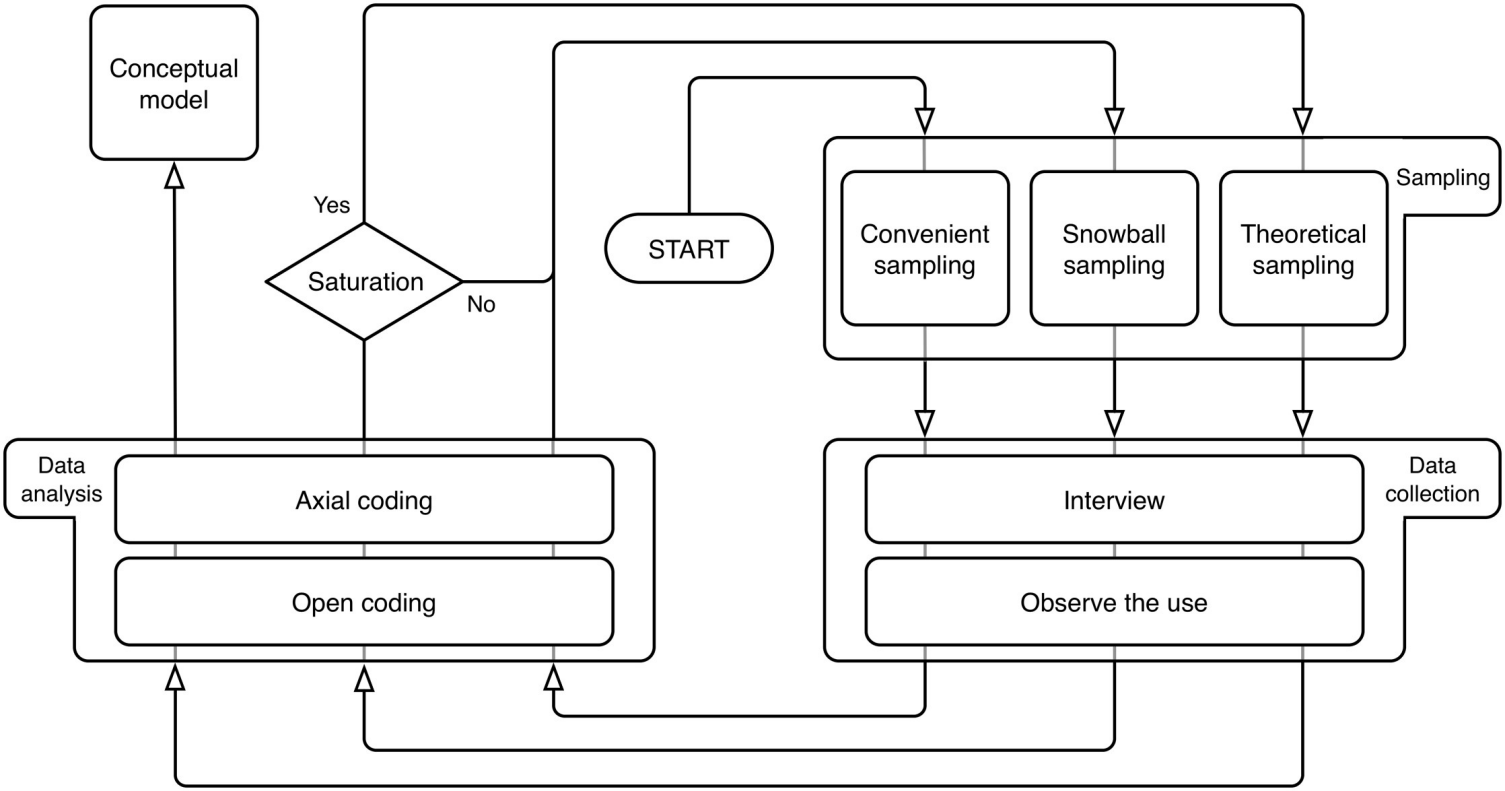
Flow diagram of the GT process. The process consists of three phases: *Sampling*, *Data Collection*, and *Data Analysis*. Selection of volunteers is performed during the *Sampling* phase. Relevant information about the volunteers is acquired during the *Data Collection* phase. The information is classified and codified during the *Data analysis* phase. The *Sampling*–*Data Collection*–*Data Analysis* cycle repeats until no more information is gained in the process (saturation). The activities of each phase changes as the cycle evolves. See the text for more details.

This methodology involves three phases: sampling, data collection, and data analysis [31]. The development of each phase proceeds iteratively and simultaneously, and are described below. The phases, processes, and steps of the Grounded Theory methodology are summarized in Fig 1.

#### Sampling phase

The sampling phase is performed following three processes [31]: convenient, snowball, and theoretical sampling.

First, during convenient sampling; we choose a set of representative individuals, selecting volunteers that have different characteristics between them, with the objective of encompassing a wide range of user profiles.

Next, using snowball sampling; new individuals who present at least one of the characteristics established as relevant are selected. Relevant characteristics are those most often repeated among the volunteers that presented a high number of problems during convenient sampling.

Finally, when the first signs of data saturation are revealed, the theoretical sampling stage starts. During this stage, data are collected, coded and analyzed in order to generate a theory. Saturation of data is the point at which no new information is extracted from the data. [32]. In GT, it is unnecessary to define a number of samples before starting the study, since this number is established when the data analysis shows a tendency to the reduction of new results (ideally close to zero). This is known as data saturation, and when the data meets this condition, the sampling number is reached.

#### Data collection phase

The data collection phase aims to acquire all the information required to identify objective and subjective characteristics relative to the phenomenon to be studied. This phase comprises three stages [31]: The first stage corresponds to analysis of documents relative to the phenomenon. The outcome of this stage is a set of activities to be performed by the subjects and a questionnaire to be used in the next stage. This questionnaire allows the evaluation of key factors that could impact the performance of the subjects during the activities. The second stage corresponds to an interview with the subjects based on the questionnaire design in the previous stage, in order to gather prior information relevant to the investigation. Finally, the third stage corresponds to the observation of the subjects by the researcher while they carry out the activities defined above.

#### Data analysis phase

Data analysis corresponds to the last phase of the methodology and includes two coding steps [31, 33]. During the coding process, the transcribed data are organized into categories and then similar categories are grouped together to organize the data for the conceptual model. The process seeks to find topics, concepts, patterns, relationships and similarities through the constant comparison method [34]. We use the GT open coding and axial coding techniques to analyze the data. During open coding, categories are developed. This involves the identification of chunks or units of data (e.g. key words, phrases, sentences) that belong to or represent a more general phenomenon [35]. In this work, we call these units of data “segments of interest”, which correspond to the difficulties identified by the researcher during the observation of use. We cluster the segments of interest that represent similar situations into RDUs (Representative Data Unit) [35–37]. During axial coding, the categories which have emerged from open coding are clustered in order to organize them in a logical and simplified way [31]. At this stage, we seek to understand and establish different emerging relationships in order to group categories that show a central association. The categories obtained from the conceptual model are checked for exclusively (i.e. that there is no overlap between them) [34].

## Materials and methods

### Participants

Thirteen participants volunteered to take part in this study. The participants showed varying characteristics, encompassing a wide range of potential users of HHMD. The study was approved by the ethics committee of the Universidad de Valparaíso. Each volunteer provided written informed consent and gave permission to publish the obtained data before the session. During each session, the researcher studied the interaction of each volunteer with a multi-parameter monitor, performing a set of predefined tasks.

### Multi-parameter monitor

The medical device under study was a multi-parameter monitor (Mindray model MEC-1200), which measures five vital signs of the human body: blood pressure, heart rate, SpO2 (saturation of peripheral oxygen), respiration rate, and body temperature. The patient’s parameters are displayed in real time on the screen of the device. We selected this device because monitoring vital signs continuously and simultaneously is relevant to homecare, since there are patients with chronic diseases who do not need to remain hospitalized, but need to constantly monitor their vital signs to prevent complications of chronic diseases [38]. In addition, the use of devices that enable people to manage their health care in a more convenient and independent way is an increasing tendency [13, 39].

### Instantiation of the grounded theory

Firstly, the researcher performed the analysis of the device’s manual before initiating the sessions with the volunteers. Here, information was extracted regarding the technical and clinical characteristics of the medical device, such as the variables measured by the device, the details of the user interface, and its clinical use. Interviews with the volunteers were then performed. The interview was based on the selection of factors that may lead to an unsafe use, as described in the literature [9, 11–13, 15, 39]. These factors include: experience in the use of medical devices, technical and medical knowledge of the health condition, physical capacities (e.g. strength), cognitive capacities (e.g. concentration and memorization) and sensory capacities (e.g. vision). Characteristics which require medical diagnoses (e.g. sensory capabilities) were based on information declared by the volunteer. New questions were incorporated into the interview based on the observations of the first convenient sampling sessions. This allowed the incorporation of features that had not been considered initially, and that could influence the safety of the use of the device. These new characteristics were evaluated immediately in the next interviews. In this study, three new characteristics were added after the first convenient sampling session: “mathematical knowledge”, “knowledge of technical symbols”, and “reaction to adverse events”. Finally, the observation of the use of the medical device was performed during each session. Participants followed a guide which was based on the functions described in the device manual. This guide defined 26 task scenarios designed for evaluating the performance of each volunteer in the use of the medical device. Here, the researcher obtained information of the use of the device. For example, tasks established for this study were “Enter a new patient dataset”, “Set proper alarm volume and display alarm limits”, and “Adjust respiration: set the alarm on, set apnea alarm to 20 seconds, establish the calculation type to automatic threshold; connect the sensor to the device”.

Each session began with an introductory period in which the researcher explained the purpose of the study and the procedure of the session. Then, a training period was carried out on the use of the multi-parameter monitor. After the training period, the volunteer was asked to perform tasks established on the task guide in order to evaluate the use of the medical device. The researcher asked the participants to “think aloud” during the session to facilitate the identification of problems. While the users performed the tasks, the researcher observed and took notes of participant actions, problems, and errors.

At the end of the tasks, and in a dynamic governed by Gordon Pask’s teachback method [40], the key elements that were registered during the session were repeated to the volunteer orally to verify that they capture the idea of the situations presented.

During the coding process, the researcher identified segments of interest of the transcriptions by applying open coding. Segments of interest were clustered to represent similar situations into RDUs. For example, segments such as “How do I know which sound level is appropriate to me? I cannot hear it” or “I cannot know how loud it is, so I will put a high number to be sure to hear it well” are associated to the RDU “User does not know if he is satisfied with the selected alarm level because he cannot test it”.

**Fig 2:**
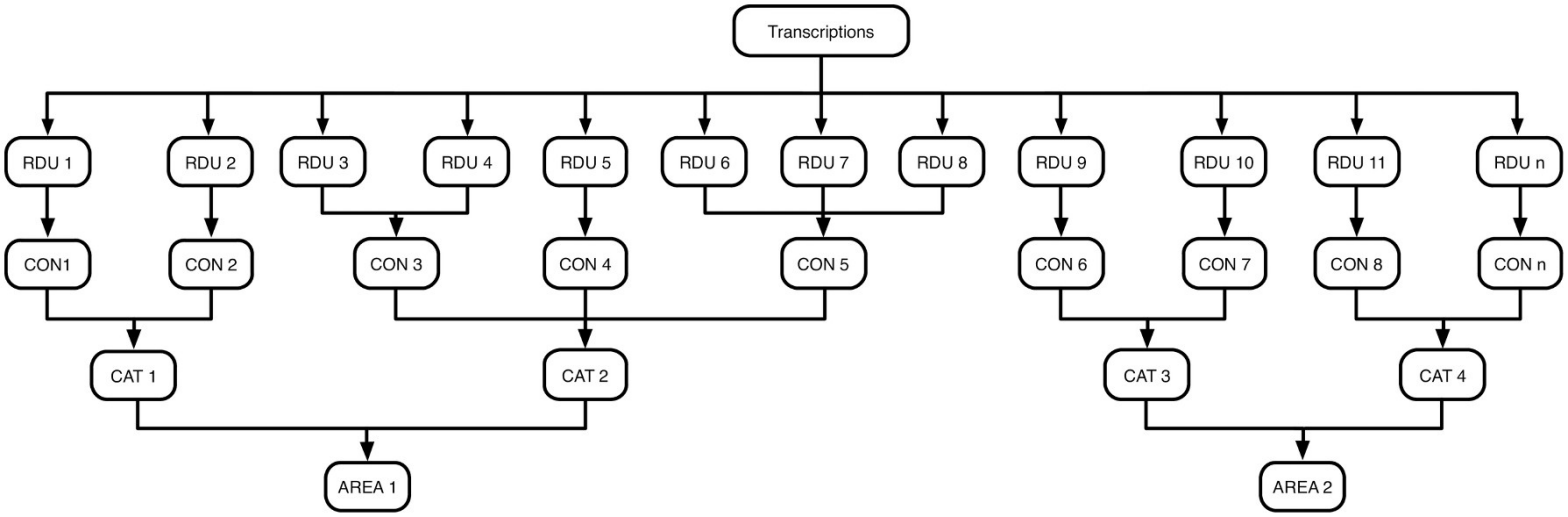
Elements obtained through coding and its relationship. The researcher transcribes fragments of the sessions with the volunteers during the open coding process. From these transcripts, the researcher distills a set of data units, called RDUs. These units are mutually exclusive, and cover the whole conceptual model. Similar RDUs are summarized in “concepts” (CON). Finally these “concepts” are clustered in categories (CAT) based on the cause of the error or problem associated to each concept.

The relationships between the elements of the conceptual model are illustrated in Fig 2. In this elicitation process, we created the RDUs from the transcripts using open coding, and created concepts from the RDUs using axial coding. Finally, concepts were grouped to create categories using axial coding. These categories constituted the conceptual model. The detection of relationships and similarities was supported by the constant comparison method [34]. The detected RDUs were encoded to represent the phenomenon. For this, we identified the error or problem which occurred in each RDU, and combined it with similar ones under a sentence that represents the problem; each cluster is called “concept” (CON). Several RDUs can be identified with the same concept [35–37]. For example, RDUs obtained in the study such as “User does not know the meaning or function of an option” and “User misunderstands the meaning or function of an option” are associated to the concept “User does not understand the function or meaning of an option or term that involves unfamiliar technical terms.”

The initial concepts were compared iteratively with each other and with the new arising concepts, focusing on finding similarities and differences, in order to create clusters of concepts. The similarities and differences were related to the cause of error or problem. As such, the concepts that present similar causes were grouped under a sentence that represents the cause; each cluster is called “category” (CAT). Each category was associated to a characteristics acquired from the device (during the manual analysis stage), user, or environment (during the interview and observation stage). The initial interpretations were modified and refined iteratively by comparing them with other segments of data and other transcripts [35–37]. For example, the category “User’s initiative to read the manual or follow the instructions” emerges from the following observed situations: “User focuses on the end of the task, not in the process, it is explained in the manual but he does not read it” and “User observes the options but does not identify the correct one, the information is in the manual but he does not read it.”

The researcher checked the categories obtained for the conceptual model, ensuring that there were no overlaps between them. It was also checked that the set of categories covered the whole spectrum of RDUs by applying constant comparison method. Afterwards, the researcher linked the created categories in order to organize them in a logical and simplified way. In this study, categories were organized in groups (according to whether the cause of error was due to the user or the device) and subgroups. In the subgroups, the categories which represented similar causes of problems were linked in a unique area. There were four areas (subgroups) created: semantics, perception, information, and manipulation.

When the saturation of the data was reached, the data analysis phase was completed, and the conceptual model created. The conceptual model represents all the categories obtained, organized into groups and areas. We represented the conceptual model by a graphical representation. This representation allows decision makers to evaluate each of the generated categories. We evaluated each category using a Likert–type scale from 1 to 5, where 1 indicated that it is not satisfied, and 5 indicated that the category fully met the established characteristic.

## Results

A total of 13 volunteers and one expert in the use of medical devices were interviewed (7 volunteers using convenient sampling, 4 volunteers using snowball sampling and 2 volunteers using theoretical sampling). This number of participants is in line with the numbers typically reported in the literature, and although in GT the necessary number of sessions for the sampling phase and for achieving saturation is uncertain, Guest et al. [32] establish that saturation is usually achieved within the first 12 interviews. We included an expert (nurse with experience in the use of multi-parameter monitors) to verify that the proposed methodology allows the detection of risks of use of medical devices by inexperienced users.

The convenient sampling phase included volunteers with different characteristics, such as a wide range of ages (23 to 67 years) and occupations (e.g. professionals, students, driver, and housewife), both genders, different levels of knowledge of technical and medical issues, with or without sensory impairment, chronic condition, or multiple language speaking, among others (see S1 Table for details). Although there were volunteers who knew this medical device, none of the volunteers had manipulated one before. Particularly, seven sessions were designed in order to detect the most interesting volunteers’ characteristics. After these sessions, we observed the relevant characteristics of the volunteers (that is, the user characteristics most often repeated among the volunteers that presented a high number of problems). The volunteers’ relevant characteristics were: people not working in the health sector, who also had some sensory impairment or chronic condition, as well as low knowledge of technical and medical issues.

**Fig 3:**
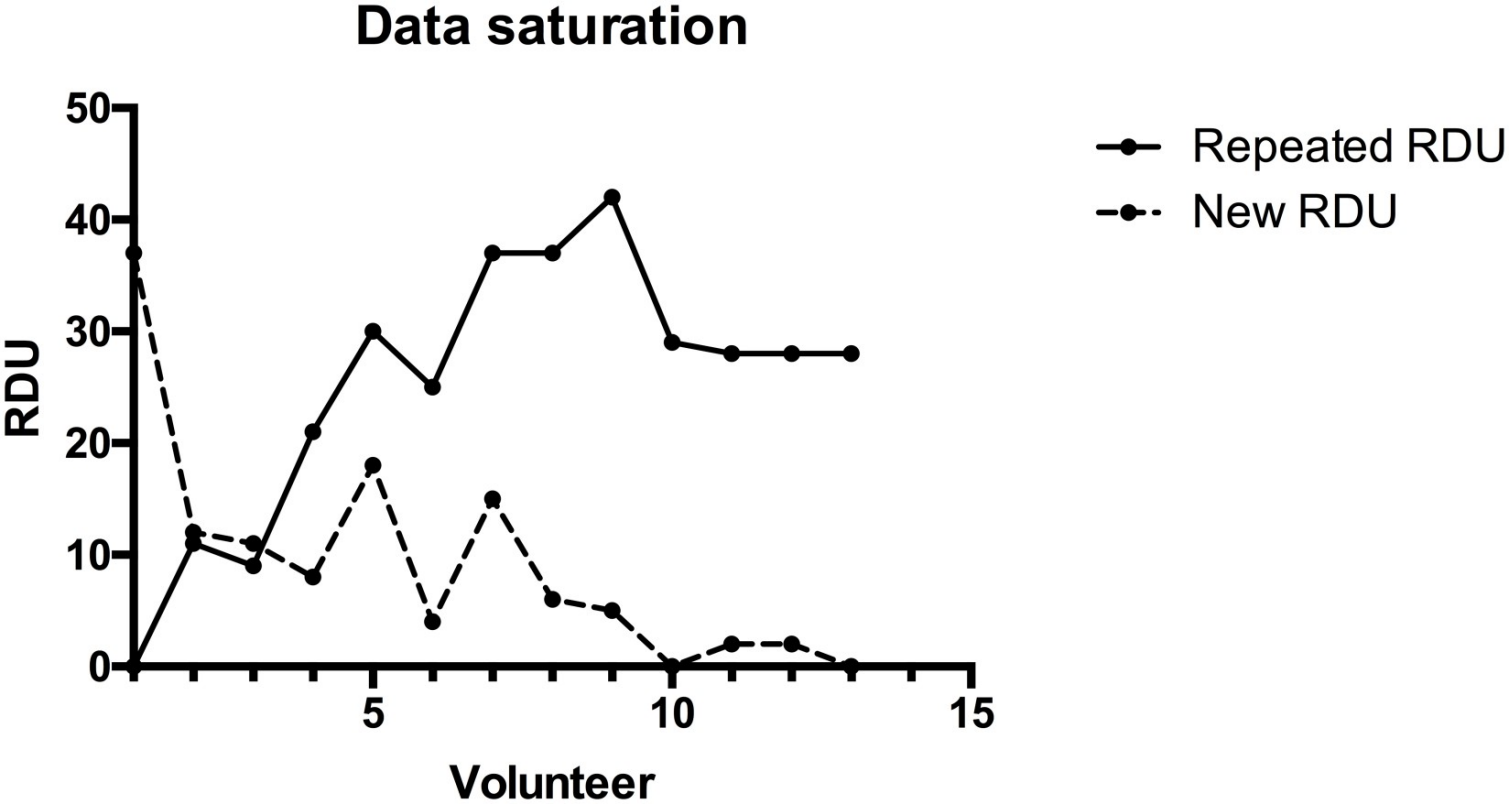
Number of new and repeated RDUs. The figure shows the evolution of the number of new (in red) and repeated RDUs (in blue) as new volunteers were added to the model. The green line represents the saturation level.

The snowball sampling phase was then initiated, including new volunteers with the relevant characteristics obtained in the convenient sampling process. The first signs of saturation were detected with the third volunteer of this sampling. Then, two sessions of theoretical sampling were needed to confirm saturation. In conclusion, the last four volunteers presented a number of new RDUs close to zero, which is the criterion to determine saturation. Fig 3 shows the saturation data graphically. We can observe that the saturation was reached within the first 12 volunteers.

During the data analysis phase, around 300 segments of interest were identified from the transcriptions of the sessions with the volunteers. From these transcriptions emerged 233 RDUs (including repeated RDUs among different volunteers). By eliminating duplicates, the list was reduced to 110 RDUs (see S2 Table for the complete list). We found that the number of RDUs did not correlated with the level of technical and medical knowledge of the volunteers. The expert user, despite not having previously manipulated the same model and brand of the device, presented a low number of difficulties (only three UDR were detected, mainly attributable to not having read the device’s manual). The most frequent RDUs are shown below. In parenthesis we indicate the percentage of participants who exhibit these RDUs.

- User does not know a medical abbreviation preventing her from understanding a task (92.3%).
- User reads the label but does not know which sensor to use to measure a specific parameter (84.6%).
- User cannot explain correctly the information of a table (76.9%).
- User does not know if she is satisfied with the selected alarm loudness because she cannot test the volume (76.9%).

All the obtained RDUs were classified in 73 concepts (types of errors or problems). The most common concepts are shown below. In parenthesis we indicate the percentage of participants who were associated with these concepts.

- User focuses on the end of the task, not in the process; this is explained in the manual but he does not read it (92.3%).
- User observes the options but does not identify the correct one, the information is in the manual but he does not read it (84.6%).
- User does not know if he is comfortable with his choice due to lack of feedback (76.9%).

Then, after a constant comparison method, we detected similarities and differences between concepts. The concepts were grouped into 30 categories (causes of error or problem). The most common categories are shown below.

- The language of the device is familiar to the user.
- User’s knowledge of technical terms of the medical device.
- User’s knowledge of medical abbreviations.
- User’s initiative to read the manual or to follow the instructions.

Table 1 shows part of the obtained RDUs, concepts and categories.

**Table 1:** RDUs, concepts and categories.

RDU Examples	Concept Examples	Categories
User does not know a medical abbreviation, which prevents him from understanding the situation.	User does not know, does not relate to, or confuses a medical abbreviation indicated in the device or manual.	Device use of language familiar to the user.
User does not know which parameter to modify to display the required data.	User is not familiar with the parameters shown in the interface.	
User does not know which option to choose, he does not know which one fits to his profile.	User does not associate the task with the options he has.	User’s knowledge of technical terms of the medical device.
User does not know how to approach the information in the manual.	User does not understand what is indicated in the manual because he is not familiar to the technical terms used in it.	
User does not know if he is satisfied with the selected alarm level because he cannot test it.	User does not know if he is comfortable with his choice for lack of feedback.	Device capacity of keeping user informed with sufficient feedback.
User does not know in which mode the device is working, it is not indicated.	User does not know in which state or mode the device is in for lack of feedback.	
User does not know how to start or cancel a measure.	User observes the options but does not identify the correct one, the information is in the manual but he does not read it.	User’s initiative to read the manual or follow the instructions.
User misses a step of the task.	User focuses on the end of the task, not in the process, it is explained in the manual but he does not read it.	
User does not notice the visual signals indicating the electrical supply is shut down.	The visual signals triggered by an adverse event do not alert the user.	Capacity of the device’s visual signals to alert user.
User cannot read the instructions.	User is not able to read the manual for the font size.	User’s visual acuity.
User cannot see the labels of buttons.	User does not know which button use, because of the label size.	

Finally, we grouped categories into areas (according to the factor associated to the cause of error). Categories presenting similar causes of problem were linked to a unique area. We determined four areas: semantics, perception, information, and manipulation.

**Semantics:** This area refers to the meaning of words. It includes concepts that are related to the gap between the terms used in the device and the terms known by the user.
**Perception:** It corresponds to what the user perceives. It includes concepts that are related to the gap between what the user actually senses and what he should sense to use the device correctly.
**Information:** it refers to the information that the user can use and his understanding. It includes concepts related to the gap between the quality of information that the user has and the information that he needs to use the device correctly.
**Manipulation:** this area refers to the physical manipulation of the device and its accessories. It includes concepts related to the gap between manipulating the device’s controls effectively, buttons and accessories and errors in their manipulation.

**Fig 4:**
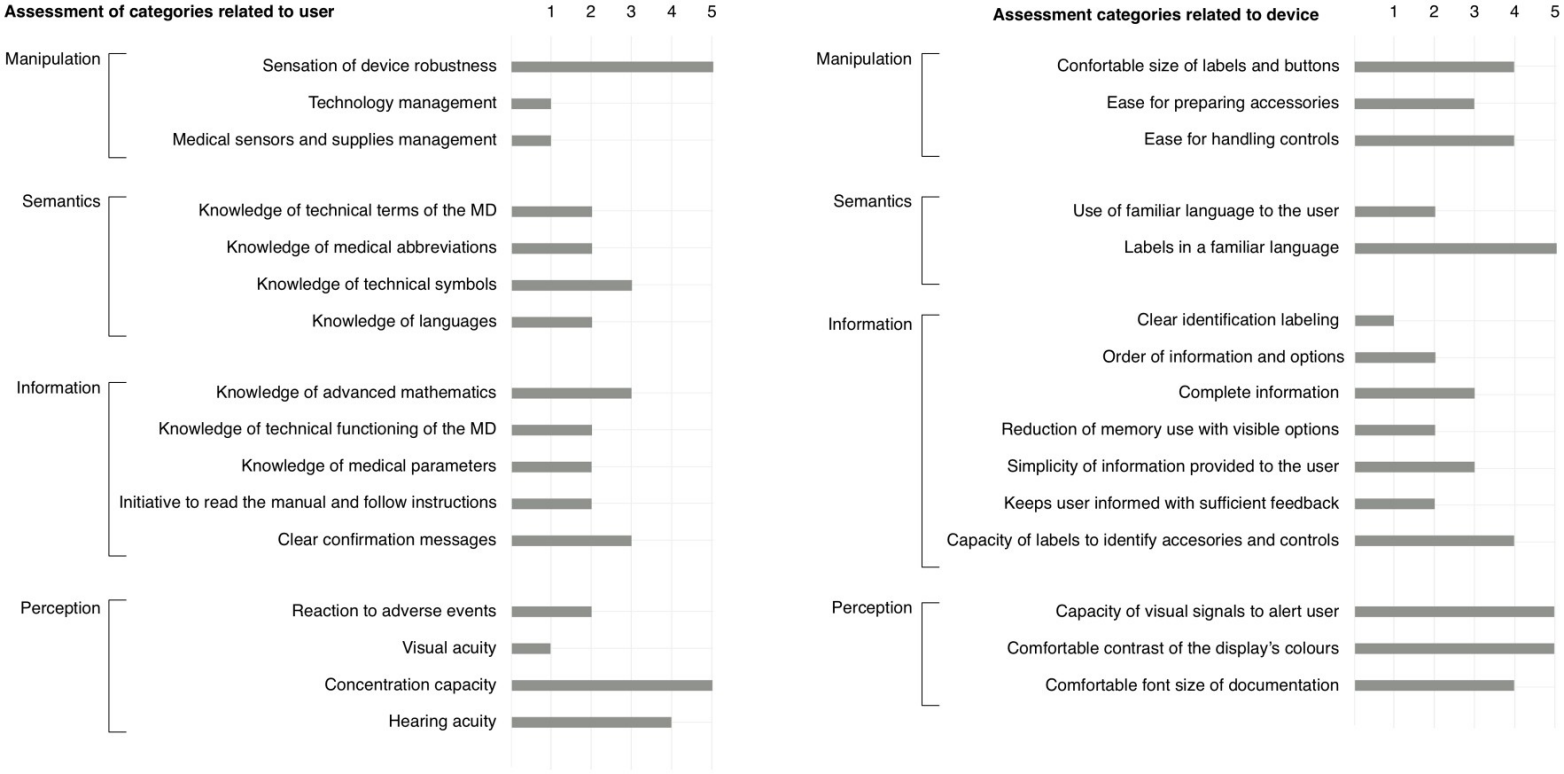
Visualization of the conceptual model. The figure shows the 30 identified categories and a corresponding score from 1 (the category is not satisfied) to 5 (the category is fully satisfied). Left column shows the categories related to the user, and the right column shows the categories related to the medical device. The categories are also grouped vertically according to the area they belong.

The conceptual model was created when saturation was reached (no more concepts created) and all categories were linked into areas. Each of these areas was classified into two groups: user, and device. Fig 4 shows categories and areas obtained through GT process.

We reify the conceptual model as a graphical representation (Fig 4). The graphical representation allows stakeholders to evaluate at glance each of the 30 categories by group and areas. The numerical evaluation was made by the researcher based on the number of RDUs that were found during the study. The score ranges from 1 (the category is not satisfied) to 5 (the category is fully satisfied). The score for each category was obtained from the number of RDUs associated with that category: The category score was inversely proportional to the number of RDUs found associated with each category and relative to the total number of RDUs that were found in the study. For example, category “comfortable contrast of the display’s colours” is well evaluated because it was associated with only 2 RDUs out of the 233. The final score was computed by mapping the obtained proportion to a Likert scale.

## Discussion

The proposed model highlights groups, areas, and categories that could lead to the unsafe operation of the device under study (low scores in Fig 4). The groups, areas, and categories that do not present risk of unsafe use are the ones that show higher scores (better evaluation) in Fig 4. This type of evaluation should focus the designer of the device on groups and categories relevant for designing a HHMD version of a multi-parameter monitor. This evaluation should also be taken into account for the training lay user in the use of the device.

Fig 4 shows a low level of compliance for most categories, corresponding to a high number of problems with the use of the device by the group of volunteers. This fact may suggest potential risks associated with the operation of the device under study.

This form of analysis can be used to analyze which group, area, or specific categories are most likely to generate a problem. In this example, the subgroup “information” is more likely to generate a problem, followed by the group “semantics”. As a consequence, those areas should be reinforced to reduce the probability of unsafe operation.

The model helps to specify areas which represent (a) the weaknesses of the device under study (either in the device interface, enclosure, or user manual); and (b) the weaknesses of the user. These weaknesses may lead to errors and therefore, to an unsafe operation. As consequence, these areas must be strengthened.

The methodology proposed in this work may be applied to other devices, and enable medical equipment manufacturers to detect weaknesses in the usability of their devices and instruction manuals. Furthermore, the methodology could allow health care professionals to identify the most appropriate device for a patient by visualizing the gap between a safe operation of the device and the operation that the user will likely perform.

In order to use this methodology, we investigated volunteers with no experience in the use of the medical devices, regardless of their different technical and medical knowledge. We also found that an expert in the use of such a device produced too few RDUs to create a conceptual model. Therefore, it may not be possible to generate a conceptual model using experts.

In relation to the “Applying Human Factors and Usability Engineering to Medical Devices” FDA guide [28], we believe that our methodology has a greater emphasis on the analysis of device usage by users, and provides a step-by-step method for obtaining and evaluating risk characteristics grounded on the use of the device by lay users.

## Conclusion

This work presents a usability study to detect problems and errors related to the use of a medical device by lay users in their home environment. The collected data was analyzed according to the Grounded Theory methodology. This analysis produced a conceptual model. Finally, the conceptual model was reified as a graphical representation.

The conceptual model and its graphical representation allow stakeholders to visualize areas of interest and categories that can lead to an unsafe operation of the medical device under study. Also, following the proposed methodology, the manufacturers could detect the principal factors that may lead to an unsafe operation of their devices, and allows healthcare professionals to determine the most appropriate device for a patient in terms of its safety. It may also be useful to identify weak aspects of the device operation, and to complement these aspects with clearer instructions. As such, the methodology used for this research is appropriate for studying the usability of a medical device to be used by a lay user in a nonclinical environment.

## Supporting information

S1 Table User characteristics from interview. Table with the list of questions to determine the user characteristics, and the corresponding answers for each volunteer.
(DOCX)

S2 Table List of RDUs. Table with the list of 110 RDUs.
(DOCX)

## References

[1] Cheng M. Medical Device Regulations, Global Overview and Guiding Principles. Geneva: World Health Organization; 2003.

[2] Treu D. Reengineering Hemodialysis for the Home Environment. Home Healthcare Horizons. 2010; p. 73–78. 22049613

[3] Bayer K, Mitchell L, Gardner S, Pine R. Engaging stakeholders in the home medical device market (1st ed.). BSI Standards Ltd. Retrieved from; 2014. Available from: http://www.bsigroup.com/meddev/LocalFiles/en-US/Whitepapers/bsi-medical-device-home-devices-whitepaper.pdf.

[4] Morris M. 2016 Global health care outlook: Battling costs while improving care. Deloitte; 2016. Available from: http://www2.deloitte.com/content/dam/Deloitte/global/Documents/Life-Sciences-Health-Care/gx-lshc-2016-health-care-outlook.pdf.

[5] FDA. Medical Device Home Use Initiative. Food and Drug Administration; 2010.

[6] Research TM. Global Home Healthcare Market (By Device Types: Diagnostics and Monitoring Devices, Therapeutic Home Healthcare Devices, Mobility Assist Devices and Medical Supplies; By Services: Rehabilitation, Telehealth and Telemedicine, Respiratory Therapy, Infusion Therapy and Unskilled Home Healthcare Services)—Industry Analysis, Size, Share, Growth, Trends and Forecast, 2014—2020 (1st ed.). Transparency Market Research. Retrieved from; 2014. Available from: http://www.transparencymarketresearch.com/home-healthcare-market.html.

[7] FDA. Design Considerations for Devices Intended for Home Use. Food and Drug Administration; 2014.

[8] Moorman B. Service Models for Remote Healthcare Monitoring Systems. Home Healthcare Horizons. 2010; p. 64–68. 22049611

[9] Kaufman-Rivi D, Collins-Mitchell J, Jetley R. Design Considerations for Medical Devices in the Home Environments. Home Healthcare Horizons. 2010; p. 21–26. 22049603

[10] Commission E. Proposal for a Regulation of the European Parliament and of the Council on Medical Device, and Amending Directive 2001/83EC, Regulation (EC) No 178/2002 and Regulation (EC) No 1223/2009. Brussels.: European Commission; 2012.

[11] Weick-Brady MD, Lazerow RN. Medical Devices: Promoting a safe migration into the home. Home Healthcare Now. 2006;24(5):298–304. 10.1097/00004045-200605000-00006 10.1097/00004045-200605000-00006 16699341

[12] Bitterman N. Design of Medical Devices—A Home Perspective. European Journal of Internal Medicine. 2011;22:39–42. 10.1016/j.ejim.2010.09.017 21238891 10.1016/j.ejim.2010.09.017

[13] Follette M. Medical Devices in Home Health Care. The Role of Human Factors in Home Health Care. Workshop Summary:. 2010; p. 145–172.

[14] Henriksen K, Joseph A, Zayas-Cabán T. The Human Factors of Home Health Care: A Conceptual Model for Examining Safety and Quality Concerns. Journal Patient Safety. 2009;5:229–236. 10.1097/PTS.0b013e3181bd1c2a 10.1097/PTS.0b013e3181bd1c2a 22130216

[15] Milamed D, Lasthaus H, Hedley-Whyte J. Highlights of the New International Standard on Home Healthcare Devices. Home Healthcare Horizons. 2010; p. 27–31. 22049604

[16] IEC. Medical electrical equipment—Part 1-11: General requirements for basic safety and essential performance—Collateral standard: Requirements for medical electrical equipment and medical electrical systems used in the home healthcare environment. International Electrotechnical Commission; 2010. IEC 60601-1-11:2010.

[17] Laboratories U. Home Healthcare Equipment: An Overview. Underwriters Laboratories; 2010.

[18] Kaufman D, Weick-Brady M. Homenet: Ensuring Patient Safety with Medical Device use in the Home. Home Healthcare Nurse. 2009;27:301–307. 10.1097/01.NHH.0000356782.00998.b3 10.1097/01.nhh.0000356782.00998.b3 19448498

[19] ECRI. Top 10 Health Tecnology Hazards for 2012. Emergency Care Research Institute; 2011.

[20] Belden P, Grayson R, Barners J. Defining and Testing EMR Usability: Principles and Proposed Methods of EMR Usability Evaluation and Rating. Healthcare Information and Management Systems Society; 2009.

[21] Nielsen J, Molich R. Heuristic evaluation of user interfaces. In Proceedings of the SIGCHI conference on Human factors in computing systems. ACM; 1990. p. 249–256.

[22] Zhang J, Johnson TR, Patel VL, Paige DL, Kubose T. Using usability heuristics to evaluate patient safety of medical devices. Journal of biomedical informatics. 2003;36(1-2):23–30. 10.1016/S1532-0464(03)00060-1 14552844 10.1016/s1532-0464(03)00060-1

[23] Jaspers MWM. A comparison of usability methods for testing interactive health technologies: Methodological aspects and empirical evidence. International Journal of Medical Informatics. 2009;78(5):340–353. 10.1016/j.ijmedinf.2008.10.002 19046928 10.1016/j.ijmedinf.2008.10.002

[24] Lang AR, Martin JL, Sharples S, Crowe JA. The effect of design on the usability and real world effectiveness of medical devices: a case study with adolescent users. Applied ergonomics. 2013;44(5):799–810. 10.1016/j.apergo.2013.02.001 23453773 10.1016/j.apergo.2013.02.001

[25] Liljegren E. Usability in a medical technology context assessment of methods for usability evaluation of medical equipment. International Journal of Industrial Ergonomics. 2006;36(4):345–352. 10.1016/j.ergon.2005.10.004

[26] Schmettow M, Vos W, Schraagen JM. With how many users should you test a medical infusion pump? Sampling strategies for usability tests on high-risk systems. Journal of biomedical informatics. 2013;46(4):626–641. 10.1016/j.jbi.2013.04.007 23688827 10.1016/j.jbi.2013.04.007

[27] IEC. Medical devices—Application of usability engineering to medical devices. International Electrotechnical Commission; 2007. IEC 62366.

[28] FDA. Applying Human Factors and Usability Engineering to Medical Devices. Food and Drug Administration; 2016

[29] Chesebro JW, Borisoff DJ. What make qualitative research qualitative? Qualitative Research Reports in Communication. 2007;8:3–14. 10.1080/17459430701617846

[30] Glaser B, Strauss A. The Discovery of Grounded Theory: Strategies for Qualitative Research. Chicago: Aldine Publishing Company; 1967.

[31] Gupta S. Design and delivery of medical devices for home-use: drivers and challenges [phdthesis]. University of Cambridge; 2007.

[32] Guest G, Bunce A, Johnson L. How Many Interviews Are Enough? An Experiment with Data Saturation and Variability. Field Methods. 2006;18:59–82. 10.1177/1525822X05279903

[33] Calman L. What is Grounded Theory? [phdthesis]. University of Manchester; 2006.

[34] Au JS, Taylor G, Newton EW. Grounded Design Theory of Japanese Fashion Designers The Design Journal. 2003;6:3–23. 10.2752/146069203789355309

[35] Spiggle S. Analysis and Interpretation of Qualitative Data in Consumer Research. Journal of Consumer Research. 1994;21:491–503. 10.1086/209413

[36] Strauss A, Corbin J. Basics of Qualitative Research: Techniques and Procedures for Developing Grounded Theory. London: Sage Publications; 1998.

[37] Goulding C. Grounded Theory: Some Reflections on Paradigm, Procedures and Misconceptions. Wolverhampton: University of Wolverhampton; 1999.

[38] Ribeiro A, Maitelli A, Valentim R, Leite C, Guerreiro A. Wireless Monitoring of Patient´s Vital Signs. Medical Informatics. 2012; p. 137–156.

[39] Gupta S. Design and Delivery of Medical Devices for Home-use: Drivers and Challenges In: Medical Electrical Devices and Technology. London; 2007 p. 215–235.

[40] Pask G, Kallikourdis D, Scott BC. The Representation of Knowables. International journal of man-machine studies. 1975;7:15–134. 10.1016/S0020-7373(75)80003-4

