# Supplementary material for: Towards a conceptual model for the use of home healthcare medical devices: The multi-parameter monitor case
Source: PLoS One. 2018 Dec 7;13(12):e0208723. doi: 10.1371/journal.pone.0208723 (PMC6285365; doi:10.1371/journal.pone.0208723)
Supplement: S1 Table — Table with the list of questions to determine the user characteristics, and the corresponding answers for each volunteer. (DOCX) [file pone.0208723.s001.docx]

**S1 Table.** User characteristics from interview.

|  | V1 | V2 | V3 | V4 | V5 | V6 | V7 | V8 | V9 | V10 | V11 | V12 | V13 |
| --- | --- | --- | --- | --- | --- | --- | --- | --- | --- | --- | --- | --- | --- |
| User characteristic |  |  |  |  |  |  |  |  |  |  |  |  |  |
| Speak only Spanish | NO | NO | NO | NO | YES | NO | YES | YES | NO | NO | NO | NO | NO |
| Has no experience in the use of medical devices | NO | NO | NO | NO | NO | YES | YES | YES | YES | NO | YES | YES | YES |
| Does not use technological equipment regularly | NO | NO | NO | NO | YES | NO | NO | YES | YES | NO | NO | YES | NO |
| Physical capacities diminished (force, dexterity, coordination, resilience) | NO | NO | NO | NO | NO | NO | YES | NO | YES | NO | NO | NO | NO |
| Attention or concentration capacity diminished | NO | NO | NO | YES | YES | NO | NO | NO | NO | NO | NO | NO | NO |
| Reading comprehension capacity diminished | NO | NO | NO | NO | NO | NO | NO | NO | NO | NO | NO | NO | NO |
| Memorization capacity diminished | NO | NO | NO | NO | NO | NO | NO | NO | NO | NO | NO | NO | NO |
| Visual deficiency | YES | NO | NO | NO | YES | YES | YES | YES | NO | YES | YES | NO | YES |
| Auditory deficiency | NO | NO | NO | NO | NO | NO | NO | NO | NO | NO | NO | YES | NO |
| Sensitivity alteration | NO | NO | NO | NO | NO | NO | NO | NO | NO | NO | NO | NO | NO |
| Confusion, overwhelm, or stress when learning new technologies | NO | NO | NO | NO | YES | NO | YES | NO | NO | NO | NO | NO | NO |
| Feeling of overwhelm in case of using a HHMD | NO | NO | NO | NO | NO | NO | NO | NO | NO | NO | NO | NO | NO |
| Low or medium knowledge of technical terms about the MD | YES | YES | YES | YES | YES | YES | YES | YES | YES | YES | YES | YES | YES |
| Low or medium knowledge of technical working of the MD | YES | YES | YES | YES | YES | YES | YES | YES | YES | YES | YES | YES | YES |
| Low or medium knowledge of medical or technical acronyms. | YES | YES | NO | YES | YES | YES | YES | YES | YES | YES | YES | YES | YES |
| Low or medium knowledge of medical parameters | YES | YES | NO | NO | NO | YES | YES | YES | YES | YES | YES | YES | YES |
| Low or medium knowledge of medical sensors and actuators | YES | YES | NO | NO | YES | YES | YES | YES | YES | YES | YES | YES | YES |
| Mathematical knowledge | - | - | YES | YES | YES | NO | YES | YES | YES | NO | YES | YES | NO |
| Knowledge of technical symbols | - | - | - | - | - | - | YES | NO | YES | NO | YES | NO | NO |
| Bad reaction to adverse events | - | - | - | - | YES | YES | YES | NO | NO | YES | NO | NO | NO |
| Does not read the user manual by its own initiative | YES | YES | YES | YES | YES | YES | YES | YES | YES | YES | YES | YES | YES |
| Considers the MD as fragile | YES | NO | NO | YES | NO | NO | NO | NO | NO | NO | NO | YES | NO |
