# Supplementary material for: Towards a conceptual model for the use of home healthcare medical devices: The multi-parameter monitor case
Source: PLoS One. 2018 Dec 7;13(12):e0208723. doi: 10.1371/journal.pone.0208723 (PMC6285365; doi:10.1371/journal.pone.0208723)
Supplement: S2 Table — Table with the list of 110 RDUs. (DOCX) [file pone.0208723.s002.docx]

**S2 Table.** List of RDUs.

| RDU |
| --- |
| Usuario no sabe qué opción elegir, no sabe cuál es la que se ajusta a su perfil |
| Usuario se complica al pedirle que deduzca un dato a poner en vez de entregarle un dato |
| Usuario no diferencia cuando se le pide que seleccione entre una opción y una tecla |
| Usuario no sabe qué opción elegir en la interfaz para realizar una tarea |
| Usuario duda sobre el significado o funcionalidad de una opción |
| Usuario no sabe el significado o funcionalidad de una opción |
| Usuario interpreta mal el significado o funcionalidad de una opción |
| Usuario no conoce un término técnico |
| Usuario no conoce término técnico de una opción |
| Usuario no logra deducir funcionalidad de indicadores que involucran palabras técnicas |
| Usuario no conoce término técnico de indicador por lo que no sabe cómo reaccionar al respecto |
| Usuario confunde significados de términos técnicos |
| Usuario no se da cuenta que hay más información en una tabla que la que se alcanza a ver en pantalla |
| Usuario no se da cuenta que hay más información en un gráfico que la que se alcanza a ver en pantalla |
| Usuario no logra ver el gráfico formado con los datos adquiridos, está desplazado |
| Usuario selecciona una opción con nombre similar al que se le pide seleccionar |
| Usuario no encuentra la tecla solicitada en la interfaz |
| Usuario no puede explicar correctamente la información de una tabla |
| Usuario no conoce una sigla médica, lo que le impide comprender la situación |
| Usuario no relaciona una sigla médica, lo que le impide comprender la situación |
| Usuario confunde una sigla médica, lo que le impide comprender la situación |
| Usuario ingresa valor errado en parámetro que se ajusta acorde al paciente |
| Usuario no sabe qué valor ingresar en parámetro que se ajusta acorde al paciente |
| Usuario no conoce unidad de medida indicada en el dispositivo |
| Usuario no sabe qué significa el parámetro que se le pide ajustar |
| Usuario no sabe a cuanto modificar un parámetro para visualizar los datos |
| Usuario no sabe qué parámetro modificar para mostrar los datos solicitados |
| Usuario no nota la diferencia al ajustar un parámetro |
| Usuario no puede explicar correctamente la información de una tabla |
| Usuario no puede explicar correctamente la información de un gráfico |
| Usuario no conoce símbolo usado para identificar un botón |
| Usuario presiona el botón para silenciar para intentar apagar un indicador visual de error |
| Usuario no sabe dónde conectarse un sensor ya que las siglas que lo indican están en inglés |
| Usuario no puede leer las instrucciones |
| Usuario no identifica las señales visuales de corte de suministro eléctrico |
| Usuario no sabe si se produjo el cambio realizado |
| Usuario configura lo solicitado donde no debe |
| Usuario presiona un botón sin verificar si el cambio producido es el deseado |
| Usuario presenta dificultad para observar tiempo que va variando que presenta mal contraste de colores |
| Usuario no puede completar un campo ya que no ve las letras del teclado |
| Usuario no puede ver las etiquetas de los botones |
| Usuario no logra ver con claridad el etiquetado de los indicadores visuales |
| Usuario no sabe si está conforme con el nivel de alarma seleccionado porque no puede probarlo |
| Usuario no sabe en qué modo está operando el dispositivo ya que no se indica |
| Usuario no sabe si se efectuó su acción, espera respuesta para estar seguro, pero esta no existe |
| Usuario no sabe si se efectuó su acción, espera respuesta para estar seguro, pero esta no existe |
| Usuario no asocia led encendido como indicador de modo seleccionado |
| Usuario cree haber seleccionado una opción pero no fue registrada |
| Usuario no selecciona la opción que se solicita ya que asume que si tiene el led encendido es porque está seleccionado |
| Usuario apaga el dispositivo por equivocación y el usuario no identifica cuando está apagado o encendido |
| Usuario no sabe hasta dónde girar un control o perilla |
| Usuario no sabe y no es capaz de decidir si el trabajo realizado cumple con lo solicitado |
| Usuario no sabe qué tipo de alarma es la que está sonando |
| Usuario duda si al tener que configurar varios datos pedidos estos son en la misma ubicación |
| Usuario no sabe qué opción elegir en la interfaz para realizar una tarea |
| Usuario no sabe cómo iniciar o cancelar una medición |
| Usuario se equivoca en el modo de operación |
| Usuario no realiza un paso de la tarea |
| Usuario se equivoca en la opción que selecciona |
| Usuario no realiza un paso de la tarea |
| Usuario no sabe en qué parte del cuerpo colocar un sensor, no sale en el manual |
| Usuario no sabe cómo abordar la información en el manual |
| Usuario selecciona una tecla incorrecta, sale en el manual |
| Usuario no sabe qué tecla seleccionar, sale en el manual |
| Usuario no sabe qué tecla seleccionar, sale en el manual |
| Usuario no sabe cómo abordar la información en el manual |
| Usuario no sabe en qué posición ubicar una perilla para que quede en la función solicitada |
| Usuario no sabe cómo manipular un accesorio para que quede en la función solicitada |
| Usuario lee el etiquetado pero no sabe qué sensor usar para medir un parámetro |
| Usuario no comprende contenido de una tabla ya que el nombre de una columna no representa su contenido |
| Usuario no sabe qué cable usar ya que no comprende el etiquetado |
| Usuario selecciona una opción que borra lo recién ingresado |
| Usuario no sabe dónde ajustar una opción de un parámetro ya que no se encuentra junto con las otras opciones del parámetro |
| Usuario no puede modificar una opción que incluye hora o fecha que no sea coherente con la hora y fecha configurada en el dispositivo |
| Usuario no puede modificar una opción que incluye hora o fecha que no sea coherente con la hora y fecha configurada en el dispositivo |
| Usuario cree haber seleccionado una opción pero no fue registrada por falta de fuerza al presionar |
| Por cumplimiento de plazo se borra la información ingresada por el usuario |
| Usuario comete un error pero el mensaje de información mostrado no es claro o explicativo |
| Usuario no comprende el mensaje de confirmación que acepta |
| Usuario no comprende mensaje de confirmación, lo que le impide continuar con la tarea |
| Usuario olvida realizar una acción de la cual se le advirtió su importancia |
| Usuario no conoce término técnico de indicador por lo que no sabe cómo reaccionar al respecto |
| Usuario presiona el botón para silenciar para intentar apagar un indicador visual de error, no comprende la diferencia entre indicador visual y alarma |
| Usuario no comprende por qué se enciende un indicador si realizó la acción que éste indica, pero esta no estaba completa |
| Usuario no conoce la unidad de medida indicada en el manual |
| Usuario no conoce término técnico del manual |
| Usuario no comprende la razón de una instrucción y la ejecuta como le es más cómodo |
| Usuario duda de la instrucción señalada en el manual |
| Usuario no asocia el uso del mando de control con las actividades de selección, aumento o disminución |
| Usuario no comprende por qué no se selecciona la opción deseada al presionar el led que indica su selección en vez del botón para cambiar el modo |
| Usuario no sabe si girar o tirar una perilla |
| Usuario pide ayuda para unir dos cables porque teme aplicarles mucha fuerza |
| Usuario no sabe cómo unir dos cables |
| Usuario no sabe si se conectó bien un sensor |
| Usuario se conecta un sensor en posición errónea |
| Usuario no sabe cómo unir dos cables |
| Usuario no sabe si conectó bien el o los cables |
| Usuario no sabe cómo conectar un cable al dispositivo |
| Usuario no se da cuenta que realiza mal la conexión entre dos sensores |
| Usuario no sabe si unió bien dos insumos |
| Usuario no sabe si colocó un insumo en la posición correcta |
| Por cumplimiento de plazo se borra la información ingresada por el usuario |
| Usuario cree haber seleccionado una opción pero no fue registrada por falta de fuerza al presionar |
| Usuario presta atención a programa de TV y coloca mal un sensor |
| Usuario se distrae con ruido de TV y selecciona la opción equivocada |
| Usuario se acerca a la luz de la ventana para leer mejor |
| Usuario no ve la etiqueta de un indicador |
| Usuario, que se encuentra en ambiente ruidoso, pone volumen alto para estar más seguro de escuchar |
| Usuario se complica al tener que sostener el contenedor y a la vez eliminar el aire del cartucho |
| Usuario se complica al tener que mantener el contenedor a altura determinada y tener que operar el dispositivo, no sabe dónde colocarlo |
|  |
|  |
|  |
|  |
|  |
|  |
|  |
|  |
|  |
